# Civil registration and vital statistics in health systems

**DOI:** 10.2471/BLT.18.213090

**Published:** 2018-10-08

**Authors:** Debra Jackson, Kristen Wenz, Maria Muniz, Carla Abouzahr, Anneke Schmider, Martin W. Braschi, Nadya Kassam, Theresa Diaz, Remy Mwamba, Philip Setel, Samuel Mills

**Affiliations:** a Health Section, United Nations Children’s Fund, 3 UN Plaza, New York 10017, United States of America (USA).; b Child Protection Section, United Nations Children’s Fund, New York, USA.; c Vital Strategies, New York, USA.; d Bloomberg Data for Health Initiative, University of Melbourne, Melbourne, Australia.; e Strategic Policy UK Information Commission, London, England.; f Maternal, Newborn Child and Adolescent Health Department, World Health Organization, Geneva, Switzerland.; g World Bank Group, Washington, DC, USA.

Over the past decades, the health sector has forged a widespread network of health facilities and community health workers who carry out key public health interventions, aiming to reach the most marginalized populations with life-saving interventions. In many countries, health networks offer untapped potential to leverage health services for the notification and registration of births and deaths.

Civil registration is defined as the continuous, permanent, compulsory and universal recording of the occurrence and characteristics of vital events, notably births and deaths, but also marriages, adoptions and divorces as provided through decree or regulation in accordance with the legal requirements in each country.^1^ Globally, around one-quarter of infants do not have their births registered.^2^ Most under-five deaths occur in the first week of life,^3^ making newborns especially vulnerable to be missed by civil registration and vital statistics systems, and less likely to have a medically certified cause of death specified. Filling this information gap is essential for improving reproductive, maternal, neonatal, child and adolescent health outcomes, policies and programmes.^4^ Strengthening government systems to generate better health and population data, disaggregated by gender and age, is necessary for measuring progress towards achieving many of the sustainable development goals.^4^

Obtaining benefits from health and civil registration and vital statistics systems requires reproductive, maternal, neonatal, child and adolescent health programmes to identify, notify and record vital events linked to a unique identifier. For this to take place, barriers such as infrastructure, legal framework and policies must be addressed to enable both sectors to capitalize on opportunities.^4^ The health records for infants, children, adolescents and women of reproductive age must include core information required by the civil registry, and health workers must be aware of their responsibilities and of the importance of birth and death registration. Also, both the civil registration and vital statistics and health systems must be able to share data, while also protecting privacy.

## Health sector collaboration

There is no substitute for a fully functioning civil registration and vital statistics system. Data generated by such systems can be used to benefit the most disadvantaged and hardest-to-reach people, but only if it is universal so that no woman or child goes uncounted.^5^ The provision of legal documents to individuals through the certification of vital events is the fundamental purpose of registration systems because these documents provide legal evidence of the characteristics of the vital event, based on which governments determine rights, such as nationality, entitlements to services including health and education, and national and voter identification systems. Legal identity documents are necessary for the protection of individuals, including age-related legal provisions, such as minimum age to marry or joining the labour force.^6^

**Fig. 1:**
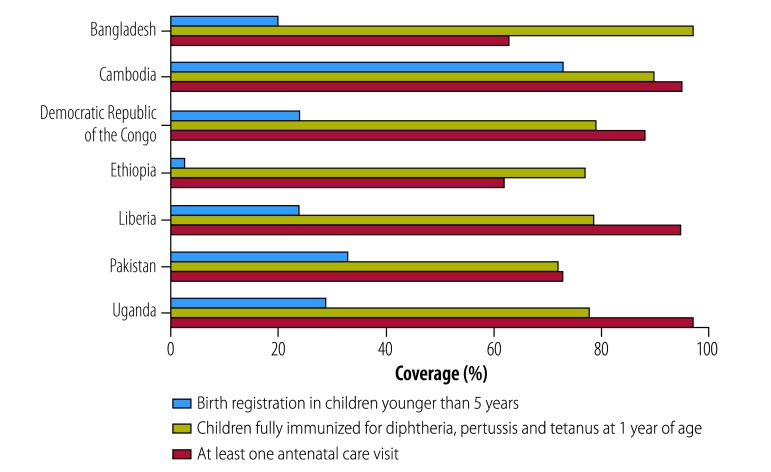
Birth registration, immunization and antenatal care coverage rates (selected countries)

Source: United Nations Children’s Fund.^7^

In many countries, the involvement of reproductive, maternal, neonatal, child and adolescent health in civil registration and vital statistics has been limited. In almost all low- and middle-income countries, birth registration coverage (children younger than 5 years whose births are registered by civil authorities) lags significantly behind key reproductive, maternal, neonatal, child and adolescent health indicators,^4^ such as coverage of antenatal care and diphtheria-pertussis-tetanus immunization (Fig. 1).^7^ One solution is to ensure that information on births and deaths (including maternal, newborn, child, adolescent and adult deaths) are notified through the health sector and submitted to the civil registry as soon as possible following occurrence of an event.

The reproductive, maternal, neonatal, child and adolescent health continuum of care, defined as integrated services for mothers and children, including both facility- and community-based services, offers an opportunity for birth and death notification and registration.^4^ Data collected during the provision of antenatal care, delivery, postpartum care, immunization and child health services, can be used both to strengthen reproductive, maternal, neonatal, child and adolescent health programmes and to generate notifications leading to registrations of birth and deaths, improving the function of the civil registration and vital statistics system (Box 1). In an example from Senegal, between 2014 and 2015, birth registration rates rose from 73% to 80%. Health workers notified, promoted and tracked birth registration, including civil registry desks within health facilities, integrating birth registration into child health booklets, and including birth registration in Senegal Child Health Day campaigns.^4^ In another programme from nine regions in the United Republic of Tanzania, health workers in health facilities have collaborated with the civil registration authority, providing both birth registration and certification during health visits, substantially increasing access to birth registration services. Between 2013 and 2017, this programme allowed registration of 1.9 million children younger than 5 years, increasing birth registration from 10.5% in 2012 to 80.4% in 2018. The United Republic of Tanzania is now extending this programme to death registration in the nine pilot districts.^8^

Box 1 **How interventions across the reproductive, maternal, neonatal, child and adolescent health continuum of care can contribute to civil registration and vital statistics^4^**

- Creating awareness of the importance of registration during antenatal and delivery care;
- Ensuring that all births and deaths that occur in health facilities are immediately notified to the civil registrar, and death notification includes cause of death;
- Increasing the potential co-location of registration facilities within hospitals and other delivery facilities;
- Notifying home births that occur with or without the assistance of skilled birth attendants or community health workers, and home deaths known to community health workers;
- Cross-checking vaccination cards or maternal and child health booklets for birth notification or registration during immunization visits for diphtheria, tetanus and pertussis vaccines and measles containing vaccine;
- Notifying unregistered home births when presented for immunization and other reproductive maternal neonatal child and adolescent health services;
- Promoting community outreach for creating demand for birth and death registration and sensitizing reproductive, maternal, neonatal, child and adolescent health service providers and skilled birth attendants on registration of births and deaths;
- Strengthening maternal and perinatal death surveillance and response systems to assure death registration and improved medical certification of causes of death; and
- Ensuring that birth and death notification forms submitted from the health sector include key information items required for the registration process.

## Systems interoperability

The interactions between health services and civil registration and vital statistics can be summarized as the interoperability between these systems, whereby civil registration services are comprehensively and consistently linked to health and other government services for mutual benefit. Interoperability is defined as the ability of information systems and procedures to share or authenticate data and enable the exchange of information and knowledge among them. This exchange is necessary to ensure cooperation, development, integration and delivery of joint services by public institutions. Stakeholders also need to implement various public policies, principles and rights, to transfer technology and to use applications that enable new services and better efficiency and cooperation among different applications.^9^

The fundamental basis of interoperability is harmonizing the systems in which information is collected, so that it is broadly reusable by a much larger set of stakeholders.^10^ Interoperability is most effective when there are mutual benefits for all stakeholders across the collaborating systems. In this example of interoperability, mutual benefits are obtained when data on births and deaths flow in both directions, from health programmes to the civil registry and vital statistics offices and back to the health programmes. The health sector notifies the civil registry of births and deaths. Civil registry data is then processed to produce vital statistics and other health data by national statistical offices. This data can then be shared back to the health sector to ensure population data is available for better targeting of services and enable databased policy and planning.

As a first step, mechanisms, legal frameworks and policies should be developed for effective communications between health workers and local registrars. This would enable immediate notification and registration for all births and deaths taking place in health facilities, as well as determination of causes of death, which are generally certified by licensed health professionals. As systems are built, this exchange of information should be a routine aspect between health and civil registration data systems.

As more health systems move to digital health management information systems, considerations for achieving civil registration and vital statistics and reproductive, maternal, neonatal, child and adolescent health interoperability has important implications for the way health data is collected, analysed and shared. Digital systems can enable real-time notifications of vital events directly to civil registries, minimizing data transmission errors and drop-offs. For this improvement in vital statistics data to take place, health sector data should shift from the collection of aggregated episode-based data to longitudinal individual records that register all encounters with health services in real time and over time. Digital solutions should be developed with government and partners in both the civil registration and vital statistics and the public health system to produce an open architecture (that is, data systems that are structured to allow data sharing) and vision that guide implementation of these solutions and innovations. Consistent standards should be used across data collection activities, within health systems and civil registration and vital statistics. Standards are needed for data sharing and interoperability between data systems. Finally, an issue that is particularly relevant for civil registration and vital statistics, with its link to legal identity systems, is to establish mechanisms to ensure data privacy and confidentiality, while enabling data sharing for analytical and public health purposes.

However, important challenges should be resolved while developing interoperable systems. Despite potential benefits to the systems, achieving interoperability is not simple. Challenges encountered from country examples include legal and governance frameworks that do not support data sharing and interoperability; public concerns about data privacy, security and patient confidentiality; inadequate information and communication technology infrastructure and connectivity; issues of server hosting and data curation; and limited human resource knowledge, capacities and skills.^4^

Overcoming these challenges requires bringing together civil registration and vital statistics and health systems stakeholders. A functional national civil registration and vital statistics steering committee to assist with the coordination of activities and collaboration and interoperability between stakeholders is key to these efforts. This collaboration should include memorandums of understanding and joint planning across ministries. Comprehensive assessment processes, particularly conducting a structured review of legal and regulatory frameworks, in addition to business process mapping to identify bottlenecks to registration, are important to any system strengthening effort.

Ensuring the survival and well-being of the most vulnerable women and children is not the role of the health sector alone. Sustained relationships between sectors such as health, civil registration, vital statistics and national identification systems are critical. Harnessing opportunities to link civil registration and vital statistics and health systems will greatly improve the availability of health services, legal identity and crucial vital statistics and population data. 
