# Corrigendum

**DOI:** 10.2471/BLT.19.100619

**Published:** 2019-06-01

In: Jackson D, Wenz K, Muniz M, Abouzahr C, Schmider A, Braschi MW, et al. Civil registration and vital statistics in health systems. Bull World Health Organ. 2018 Dec 1;96(12):861–63, 

on page 861, the name of the sixth author should be Martin W Bratschi.

